# ID 341 - Importância do Go.Data no Rastreamento de Contatos de um Caso Importado de Sarampo em Minas Gerais no Ano de 2024

**DOI:** 10.5327/2237-9622.2025.v34s1.341

**Published:** 2025-11-25

**Authors:** Lucimeire Neris Sevilha da Silva Campos, Cintia Paula Vieira Carrero, Josafá do Nascimento Cavalcante Filho do Nascimento, Luciana Oliveira Barbosa de Santana Oliveira, Lucimeire Neris Sevilha Da Silva Campos Neris, Rebeca Porto Rosa, Rita de Cássia Ferreira Lins, Nájla Soares Silva, Sara Gonzalez, Greice Madeleine Ikeda do Carmo, Marcelo Yoshito Wada, Luciene da Rocha Ribeiro, Gilmar Rodrigues, Felipe Leandro Batista

**Keywords:** sarampo, Go.Data, rastreamento de contatos, Minas Gerais, vigilância epidemiológica, 2024; eliminação do sarampo, saúde pública

## Abstract

**Introdução:**

O Brasil perdeu a certificação de país livre do sarampo em 2019, mas tem levantado esforços para recuperar o status de eliminação da doença e manter a sustentabilidade da eliminação da rubéola. Dada a alta transmissibilidade do vírus do sarampo, a resposta rápida diante de casos suspeitos é crucial para prevenir surtos. A tecnologia da ferramenta Go.Data tem se mostrado essencial no rastreamento de contatos e no monitoramento de possíveis cadeias de transmissão, contribuindo significativamente para os esforços de contenção da doença. Objetivou-se descrever a experiência do uso do Go.Data no rastreamento e no monitoramento de contatos de um caso importado de sarampo ocorrido em Minas Gerais, em 2024.

**Método:**

Analisaram-se os dados epidemiológicos da investigação realizada pela Secretaria de Saúde de Minas Gerais, que utilizou o Go.Data para monitorar os contatos de um caso confirmado de sarampo na Semana Epidemiológica 33/2024. Foram quantificados os contatos rastreados pela ferramenta e os municípios onde os contatos foram localizados.

**Resultados:**

O monitoramento ocorreu entre 31 de julho de 2024 e 26 de agosto de 2024. Durante esse período, o Go.Data registrou 300 pessoas que estiveram em contato com o caso confirmado durante o período de transmissibilidade da doença, distribuídos em nove municípios. A ferramenta permitiu o acompanhamento de todos os contatos, visualizando as possíveis cadeias de transmissão, que felizmente não ocorreram.

**Conclusão:**

O uso do Go.Data otimizou o processo de monitoramento de contatos, tarefa que, feita manualmente, seria mais demorada. Além de agilizar a coleta e a análise de dados, a ferramenta facilita a visualização de cadeias de transmissão, oferecendo uma interface intuitiva para a tomada de decisões pelos gestores de saúde. Conclui-se que o Go.Data é uma ferramenta importante para auxiliar no processo de eliminação do sarampo no Brasil, proporcionando resposta rápida e organizada diante de surtos.

